# Peste Des Petits Ruminants (PPR) in Dromedary Camels and Small Ruminants in Mandera and Wajir Counties of Kenya

**DOI:** 10.1155/2019/4028720

**Published:** 2019-03-04

**Authors:** R. N. Omani, G. C. Gitao, J. Gachohi, P. K. Gathumbi, B. A. Bwihangane, K. Abbey, V. J. Chemweno

**Affiliations:** ^1^Department of Veterinary Pathology, Microbiology and Parasitology, University of Nairobi, P.O. Box 29053, Kangemi, Kenya; ^2^Jomo Kenyatta University of Science and Technology, P.O. Box 62000-00200, Nairobi, Kenya; ^3^International Livestock Research Institute P.O. Box 30709-00100, Nairobi, Kenya; ^4^Kenya Camel Association, P.O. Box 6067-00100, Nairobi, Kenya

## Abstract

A study was conducted to determine the presence of Peste des petits ruminants (PPR) in camel population kept together with small ruminants in Isiolo, Mandera, Marsabit, and Wajir counties of Kenya. This was done in the wake of a disease with unknown etiology “*Camel Sudden Death Syndrome*” camels in the horn of Africa. Thirty-eight (38) samples, 12, 8, 15, and 3 samples, were collected from Mandera, Wajir, Isiolo, and Marsabit, respectively, from 25 camels, 7 goats, and 4 sheep. One camel in Mandera and one goat in Wajir were confirmed positive for PPR virus (PPRV) through reverse Polymerase Chain Reaction. The analysis of sequences revealed closest nucleotide identities of obtained sequences from both goat and camel to the lineage III of PPRV albeit with 60.29% of nucleotide identity. This study establishes that camels in the study area suffer with PPR manifest clinical signs that are mainly characterized by inappetence, loss of body condition, and general weakness terminally leading to diarrhea, conjunctivitis, and ocular nasal discharges preceding death. These clinical signs are similar to those observed in small ruminants with slight variations of manifestations such as keratoconjunctivitis as well as edema of the ventral surface of the abdomen. This shows that camels could be involved in the epidemiology of PPR in the region and that PPRV could be involved in the epidemics of Camel Sudden Death syndrome. There is therefore a need for resources to be dedicated in understanding the role camels play in the epidemiology of PPR and the role of the disease in Camels Sudden death syndrome.

## 1. Introduction

Peste des petits ruminants (PPR) is a virus within the genus* Morbillivirus* and in the family Paramyxoviridae that has been reported in Sub-Saharan Africa, the Arabian Peninsula, Middle Eastern countries and India mainly [1, 2]. It causes an exceedingly acute disease in domestic sheep and goats characterized by fever, lesions in the mouth, diarrhea, and pneumonia often leading to death of affected animal [1, 3–5]. The disease was first reported in Kenya in the year 2007 [6]. Of concern is that recently there have been reports on PPR causing a fatal disease syndrome in camels [7, 8].

PPR is one of the diseases that is currently targeted for eradication possibly by the year 2030 [9, 10]. PPR causes death of livestock that is highly depended on by poor households in developing nations; it is a danger to food security and international trade of livestock and livestock products [11–13]. It is therefore very important to determine presence of PPRV infection in other hosts herded with sheep and goats as this will have implication on the eradication efforts. Camels are herded together with goats in most parts of Northern Kenya. This study was aimed at investigating the presence of PPR in camels from Kenya and determining the relationship of the PPRV in camels and goats. Although camels have been documented to contract PPR disease in Sudan and Iran where camel keeping is practiced [7, 8, 14], the relationship of the virus in goats to that in camels has not been reported and neither has the condition been reported in Kenya.

## 2. Materials and Methods

**Figure 1:**
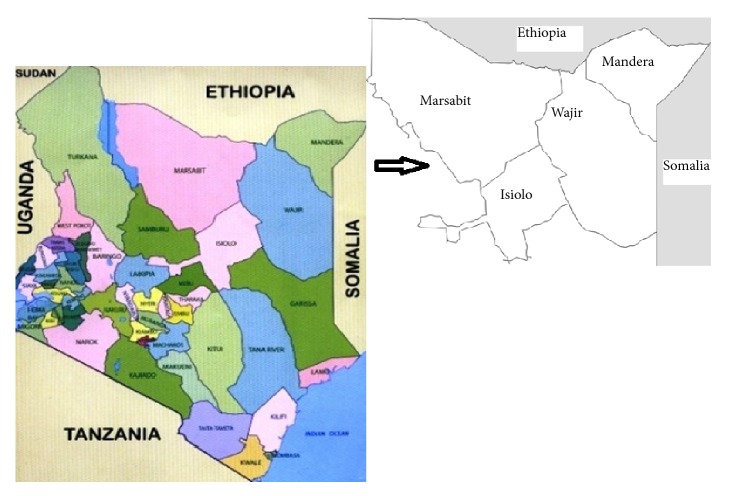
Map showing the study area.

A disease field investigation was done as from mid-February 2016 to the end of March 2016 from the study counties (Figure 1) following an outbreak of a disease of unknown etiology named “*Camel Sudden Death Syndrome*”. 36 camel herds along transport routes were purposively identified based on the recommendations of the veterinary departments in the respective counties. Three hundred and ninety-two (392) camels presented as sick by respective herders were examined for PPR. In addition 80 sheep and goats spread through 4 herds which were closely reared with camels in the same herds were also examined (See Tables 1–8 in supplementary material). The sampled animals had a fever in addition to presenting any or a combination of diarrhea, ocular, and nasal discharge.

In total, 38 biological samples (see Tables 1 and 2 in Supplementary Material), 12, 8, 15, and 3 samples, were collected from Mandera, Wajir, Isiolo, and Marsabit, respectively, from 25 camels, 7 goats, and 4 sheep. The sample included nasal and ocular discharges on viral transport media as well as EDTA blood. Samples were carried in a cool box containing icepack and stored in local laboratories in the field before they were transported to Kenya Aids Vaccine Institute, Institute of Clinical Research (KAVI-ICR) molecular laboratory where they were stored at -20°C awaiting laboratory analysis.

### 2.1. Detection of PPRV by RT-PCR

Extraction of RNA was done using QIAamp DSP Virus Kit ® as per manufacturer's (QIAGEN GmbH, 40724 Hilden, GERMANY) instructions at the KAVI-ICR. RNA quantification was further done to check for the concentration and purity of the RNA extract. The QIAamp process was used as is appropriate for blood samples that have been preserved with EDTA. RT-PCR was carried out as described by Couacy-Hyman and Ularamu [15] (Ularamu et al., 2012) (see Tables 9–11 in Supplementary Material). All reactions were run with Nigeria 75/1 vaccine strain as a positive control and nuclease-free water as the negative control. RNA extraction and RT-PCR was repeated at the International Livestock Research Institute, (ILRI) for quality control.

### 2.2. Gel Electrophoresis and Visualization

RT-PCR products were separated by electrophoresis on a 1.5% agarose gel in 0.5% TAE buffer (SERVA Electrophoresis, Heidelberg, Germany) stained with GelRed nucleic acid stain (Phenix Research Products, Candler, USA).

### 2.3. Sequencing

Samples that tested positive for PPRV using the N gene primer set were sequenced. Sequencing of the RT-PCR amplified products was done by Inqaba Biotechnical Industries (Pty) Ltd. in Pretoria, South Africa. The BLAST online tool was used to search in the GenBank for homologous gene sequences in the NCBI database with one of the sequences (see in Table 10 in Supplementary Materials).

## 3. Results

### 3.1. Disease Field Investigation

Three hundred and ninety-two (392) camels were examined from 36 herds that had a history of individuals dying suddenly from a disease of unknown etiology. The herds examined consisted of a few camels as less as three to more than 100 per herd. Ninety-three per cent 93% (367 individuals) of the camels examined although reported to have presented clinical signs typical to the disease of unknown etiology; they were not clinically diagnosed as PPR diseased camels. Those that were sampled presented with loss of body condition and general weakness, diarrhea, conjunctivitis oculonasal discharges in addition to a fever of 40 degrees. Four flocks of sheep and goats were also examined that were found herded alongside the camel herds that we examined. 13.75% out of 80 individuals identified by the herders appeared to present with clinical PPR, a fever coupled with nasal-ocular discharges and or with diarrhea.

**Figure 2:**
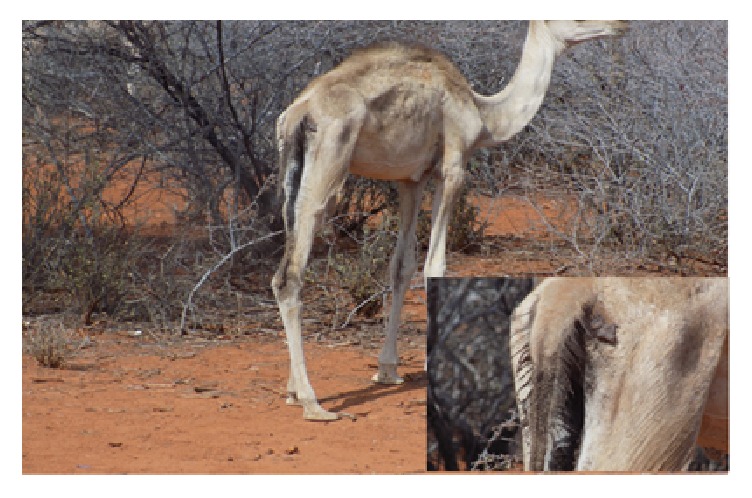
Suspected case of PPR of a male calf showing emaciation and diarrhea in Mandera.

The camel (Figure 2) was anorexic, was emaciated, had a ruffled hair coat, and had matted the perineum showing signs of diarrhea. The camel was sneezing, had nasal discharges, and had a fever of 40°C. The animal was weak and could not move in the same pace with the rest of the herd and preferred recumbency.

**Figure 3:**
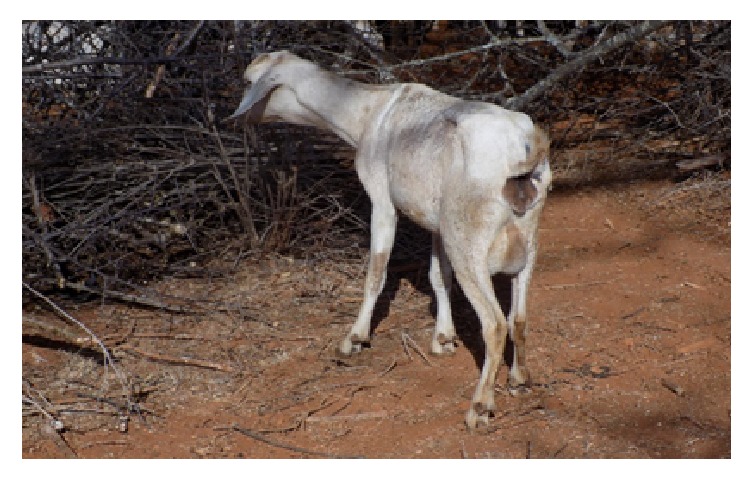
Suspect PPR case of a goat in Wajir.

This goat (Figure 3) was found among a herd of camels in a watering point in Wajir. It was anorectic and had a fever of 39.8°C with mucopurulent nasal discharges. The goat had not been treated.

### 3.2. PCR Results

**Figure 4:**
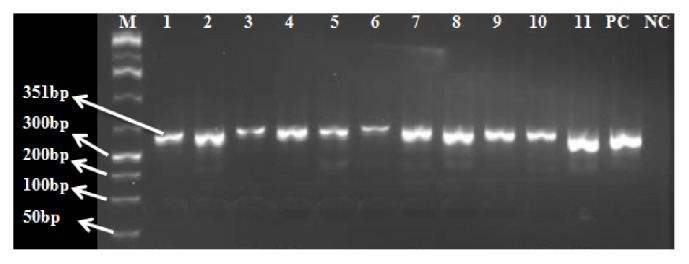
RT-PCR products visualized under UV transilluminator showing nucleoprotein gene amplicons where M is the DNA marker, 1-11 samples on gradient PCR. 1-6 is Kenya_ PPRV_ Goat _Wajir; 7-11 is Kenya_ PPRV_ Camel _Mandera, NC is negative control, and PC is positive control.

Two of the 38 samples collected turned out positive for PPRV. Using the same primers, the two samples, one from Mandera and another from Wajir, were amplified using the same primers to optimize the PCR conditions with gradient PCR machine by changing the concentration of the cDNA and the other master mix contents to yield amplicons as seen in Figure 4.

**Figure 5:**
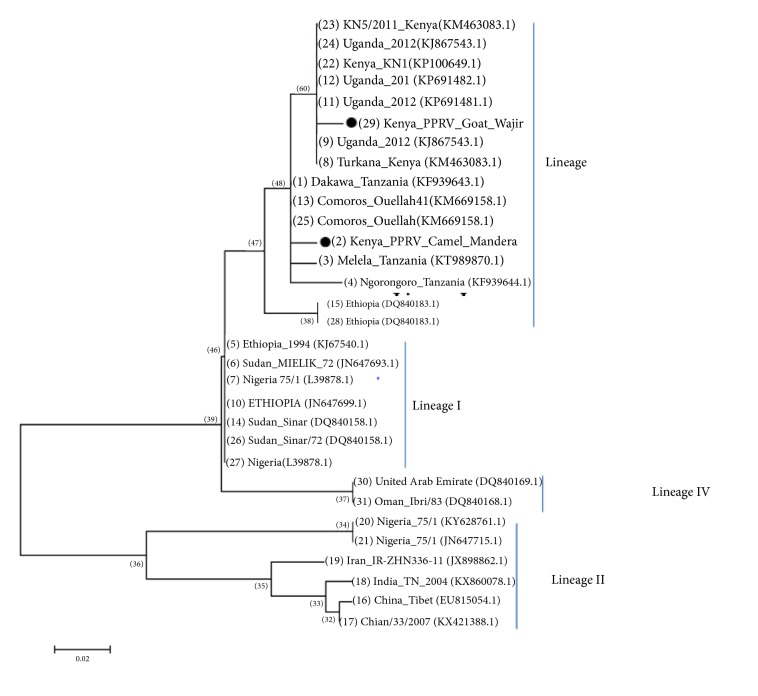
Phylogenetic tree of PPR viruses based on the N gene constructed using the neighbour-joining method in MEGA 7®software. The sample in this study is marked with a round black dot. Phylogeny was inferred following 1000 bootstrap replications.

The sequences obtained were compared to homologous gene sequences in the NCBI database (Table 12 in Supplementary Material). The analysis of sequences revealed closest nucleotide identities of obtained sequences from both goat and camel to the lineage III of PPRV found in East Africa and Ethiopia (Figure 5).

There was a 60.29% of nucleotide identity between the PPRV isolate from goat in Wajir district compare to camel isolate in Mandera in Kenya in a 351bp nucleoprotein fragment as compared using MEGA 7®software.

## 4. Discussion

PPRV is spread through contact among prone animal's species more especially sheep and goats through respired droplets, predominantly when an infected animal coughs or through excretions of clinical infective material such as nasal, ocular, and oral discharges [16–18]. Based on these dynamics the virus may be spread over areas especially through movement of infected animals especially for trade and/or during relocation for water and pastures [10, 19, 20]. This is so especially for animals that incubate the disease without overt clinical signs [5, 21].

The study has demonstrated that camels display both clinical and laboratory detected PPR. This study finding is in agreement with studies carried out in Sudan and Iran demonstrate that camels are able to develop an active clinical syndrome caused by PPRV albeit not with the same typical PPR clinical presentation in small ruminants [7, 8]. According to these findings, the affected camels present with a fatal subacute to acute syndrome loosely referred to “*Camel Sudden Death syndrome*” which is rarely noticed and if noticed is unspecific and characterized by in-appetence, loss of body condition and general weakness, diarrhea, conjunctivitis oculonasal discharges, and finally recumbency preceding death of the affected animals [7, 8]. These notable clinical signs that were encountered in this study have been encountered in camels before in the region [22, 23] and in camels that have tested positive for PPR in Iran as well as in Sudan ([7, 8].). Of notable significance; these clinical signs are more similar with those that have been observed in sheep and goats manifesting with PPR albeit with slight variations of manifestations such as keratoconjunctivitis as well as oedema of the ventral surface of the abdomen that was observed in this study as well other previous studies. The sudden onset and the fatal nature of PPR camels in the study areas and in previous literature [7, 8] also show some similarities with the disease in sheep and goat where sudden onset and high mortalities have been reported more especially in herds that have not been exposed before [9].

Lineage III of PPRV was identified in the two samples. This coincides with the lineage that has been found to be distributed in Kenya as well as other countries that are bordering Kenya including Tanzania and Uganda [6, 24–26]. This reinforces the geographic stability of this virus as stated in earlier studies that have sort to understand the genetic variation and geographical clustering of the virus [27]. This study show that although the two strains were both from lineage III, there were slight differences between the two viruses strains based on the comparative gene sequences of the two sequences obtained. Nevertheless, there was a 60.29% of nucleotide identity between the two isolates.

Of the total animals examined, 6.329% of those presented as sick had clinical signs similar to those of PPR and only one (1) of the twenty-five (25) camels had a clinical disease that was detected as PPR using laboratory signs representing only 4% of the sick population. This also occurred in sheep and goats herds where only 12.5% of the eighty individuals presented showed a clinical disease. One camel from Mandera and one goat from Wajir tested positive through RT-PCR despite the fact that all animal sampled were showing clinical signs of a disease identified as PPR. The detection of clinical* Peste des Petits Ruminants* is a challenge as there are other diseases that present with clinical signs that appear as PPR [15, 16]. Furthermore, PPR is often found as a mixed infection and also there are other diseases that present with fever and with wholly comparable clinical signs, more particularly when it is newly introduced in a herd [5, 28, 29]. Despite the low detection probability, results of on a single individual in a herd can be used to build inferences on the way the disease behaves in a herd [30]. Nevertheless, the low detectability of the disease through RT-PCR may have been attributed to the sampled animals having very low viral load hence not sensed by the assay, or the RNA might have been lost during sample processing [31]. In addition, the low detectability could also have been attributed to the sampled animals suffering from other diseases that present in the same way as PPR [5, 15, 19].

Camels have been proven to be susceptible to PPRV; nevertheless, there is limited information on their contribution in maintaining the disease in the animal population as well as in developing active and fatal disease as their small ruminants' counterparts not until recently [7, 32–34] (Zakian et al. 2014). These study findings suggest that there is possible interaction of this virus between small ruminants and camel population in Kenya subsequently necessitating the evaluation of the role enjoyed by different strains than differences in virulence and their transmission potential [27]. The clinical findings as well as the molecular tests confirm this and these correlate with the other studies that have been undertaken in some pastoral regions where small ruminants and camels share the same ecosystem [33] (Zakian et al. 2014). It was not however possible to determine if there was actual transmission of the disease between the host as the sample size was limited and temporally dispersed. It is also possible that perhaps this is the strain of lineage III that causes active infections in camel herds within the region it was isolated.

## 5. Conclusion

Emergence of PPRV in unusual hosts is a current ongoing discussion and there is need for long-term forthcoming studies especially in areas where PPRV is enzootic to understand the dynamics of the virus. Based on the findings of this study, the author would wish to recommend that, during outbreaks of PPR, camels, sheep, and goats herds should be reared separately. There is also a need for resources to be dedicated in understanding the role camels play in this disease more important especially when considering the global objective of eliminating PPR by 2030 by the OIE and the Food and Agriculture Organization (FAO).

## Data Availability

Any additional data is available upon request through the corresponding author, Dr. Ruth Omani, through ruthomani2010@students.uonbi.ac.ke; ruthomani2010@gmail.com.

## Additional Points

The data available for this study was limited and it was not possible to build inferences of the detection probabilities of the disease in the population. The study is also dependent on one laboratory method to confirm the presence of the disease in the study population.

## Supplementary Materials

Supplementary Materials List of tables that contain data of samples collected with their respective locations, RNA quantification, and homologous gene sequences from the NCBI used to form the phylogenetic tree.
