# Supplementary material for: Peste Des Petits Ruminants (PPR) in Dromedary Camels and Small Ruminants in Mandera and Wajir Counties of Kenya
Source: Adv Virol. 2019 Mar 4;2019:4028720. doi: 10.1155/2019/4028720 (PMC6425320; doi:10.1155/2019/4028720)
Supplement: Supplementary Materials — List of tables that contain data of samples collected with their respective locations, RNA quantification, and homologous gene sequences from the NCBI used to form the phylogenetic tree. [file 4028720.f1.zip › 4028720.f1/Table 1 Sample collected from tentative cases of PPR_AV_2677389.docx]

Table 1 Sample collected from tentative cases of PPR

| **BLOOD SAMPLES** | **LAB CODE** | **SPECIES** | **SAMPLE TYPE** |
| --- | --- | --- | --- |
| Mn/29/01 | 1 | Camel | Blood |
| Mn/16/01 | 2 | Camel | Blood |
| Mn/ 65/01 | 3 | Camel | Blood |
| Mn/08/01 | 4 | Camel | Blood |
| Mn/15/01 | 5 | Camel | Blood |
| Mn/14/01 | 6 | Camel | Blood |
| Mn/81/01 | 7 | Camel | Blood |
| Wj /goat/01 | 8 | Goat | Blood |
| Wj /goat/02 | 9 | Goat | Blood |
| Wj /sheep/01 | 10 | Sheep | Blood |
| Mn/65/01 | 11 | Camel | Nasal |
| Wj/goat/02 | 12 | Goat | Nasal |
| Mn/65/01 | 13 | Camel | Ocular |
| Wj/goat/01 | 14 | Goat | Ocular |
| Wj/goat/01 | 15 | Goat | Nasal |
| Wj/goat/04 | 16 | Goat | Ocular |
| Mn/goat/01 | 17 | Goat | Nasal |
| Mr/13/01 | 18 | Camel | Nasal |
| Mr/63/01 | 19 | Camel | Nasal |
| Mr/29/01 | 20 | Camel | Nasal |
| Wj/sheep/03 | 21 | Sheep | Nasal |
| V/revival/G | 22 | Goat Kid | Nasal |
| Migwi/G3 | 23 | Goat Kid | Nasal |
| Is/42/1 | 24 | Camel | Blood |
| Is/35/1 | 25 | Camel | Blood |
| Is/40/1 | 26 | Camel | blood |
| Is/15/1 | 27 | Camel | Blood |
| Is/25/1 | 28 | Camel | Blood |
| Is/12/1 | 29 | Camel | Blood |
| Is/37/1 | 30 | Camel | Blood |
| Is/36/1 | 31 | Camel | Blood |
| Is/23/1 | 32 | Camel | Blood |
| Is/18/1 | 33 | Camel | Blood |
| Is/31/1 | 34 | Camel | Blood |
| Is/41/0 | 36 | Camel | Blood |
| Is/19/0 | 37 | Camel | Blood |
| Is/27/1 | 38 | Camel | Blood |
