# Supplementary material for: Peste Des Petits Ruminants (PPR) in Dromedary Camels and Small Ruminants in Mandera and Wajir Counties of Kenya
Source: Adv Virol. 2019 Mar 4;2019:4028720. doi: 10.1155/2019/4028720 (PMC6425320; doi:10.1155/2019/4028720)
Supplement: Supplementary Materials — List of tables that contain data of samples collected with their respective locations, RNA quantification, and homologous gene sequences from the NCBI used to form the phylogenetic tree. [file 4028720.f1.zip › 4028720.f1/Table 10 List of primers used in the study_AV_2677398.docx]

*Table 10 List of primers used in the study.*

| **Name** | **Gene** | **Location** | **Sequence** | **Amplicon**  **Size** | **Reference** |
| --- | --- | --- | --- | --- | --- |
| NP3-forward | Nucleoprotein | 1232-1255 | 5’- TCTCGGAAATCGCCTCACAGACTG -3’ | 351bp | Ularamu *et al*., 2012;  Couacy-Hymann *et al*, 2002 |
| NP4-reverse | Nucleoprotein | 1583-1560 | 5’- CCTCCTCCTGGTCCTCCAGAATCT -3’ | 351bp |  |
