# Supplementary material for: Peste Des Petits Ruminants (PPR) in Dromedary Camels and Small Ruminants in Mandera and Wajir Counties of Kenya
Source: Adv Virol. 2019 Mar 4;2019:4028720. doi: 10.1155/2019/4028720 (PMC6425320; doi:10.1155/2019/4028720)
Supplement: Supplementary Materials — List of tables that contain data of samples collected with their respective locations, RNA quantification, and homologous gene sequences from the NCBI used to form the phylogenetic tree. [file 4028720.f1.zip › 4028720.f1/Table 11 RNA content quantification in sample after RNA extraction_AV_2677399.docx]

Table 9 RNA content quantification in sample after RNA extraction.

| **Sample ID** | **Nucleic A Conc./** ng/µl | **A260** | **A280** | **260/280** | **260/230** | **Sample Type** | **Factor** |  |
| --- | --- | --- | --- | --- | --- | --- | --- | --- |
| Mn/29/01 | -0.2 | -0.006 | -0.014 | 0.41 | 0.4 | RNA | 40 | Blood |
| Mn/65/01 | 1.2 | 0.03 | 0.017 | 1.73 | 0.3 | RNA | 40 | Blood |
| Mn/08/01 | 0.3 | 0.007 | -0.003 | -2.07 | 0.02 | RNA | 40 | Blood |
| Mn/15/01 | 0 | 0.001 | -0.006 | -0.18 | 0.01 | RNA | 40 | Blood |
| Mn/14/01 | 1.2 | 0.029 | 0.01 | 2.91 | 0.09 | RNA | 40 | Blood |
| Mn/81/01 | 1.2 | 0.029 | 0.016 | 1.79 | 0.08 | RNA | 40 | Blood |
| Wj /goat/01 | 10.1 | 0.254 | 0.131 | 1.93 | 0.1 | RNA | 40 | Blood |
| Wj /goat/02 | 0 | -0.001 | 0 | 1.14 | 0 | RNA | 40 | Blood |
| Wj/sheep/01 | 0.5 | 0.013 | 0.002 | 8.47 | 0 | RNA | 40 | Blood |
| Mn/65/01 | 0.7 | 0.017 | 0.005 | 3.13 | 0.02 | RNA | 40 | Blood |
| Wj/goat/02 | 0.3 | 0.008 | -0.008 | -0.97 | 0 | RNA | 40 | Nasal |
| Mn/65/01 | 0.5 | 0.014 | -0.002 | -8.07 | 0.02 | RNA | 40 | Nasal |
| Wj/goat/01 | 0.6 | 0.015 | -0.002 | -7.38 | 0.01 | RNA | 40 | Ocular |
| Wj/goat/01 | 0.2 | 0.005 | -0.004 | -1.17 | 0.04 | RNA | 40 | Ocular |
| Wj/goat/04 | 0.3 | 0.006 | -0.008 | -0.84 | 0.01 | RNA | 40 | Nasal |
| Wj/goat/01 | 3.4 | 0.085 | 0.097 | 0.87 | 0.08 | RNA | 40 | Ocular |
| Mr/13/01 | -15.7 | -0.392 | -0.205 | 1.91 | -0.06 | RNA | 40 | Nasal |
| Mr/63/01 | -13.8 | -0.346 | -0.181 | 1.9 | -0.04 | RNA | 40 | Nasal |
| Mr/29/01 | -10 | -0.25 | -0.151 | 1.66 | -0.02 | RNA | 40 | Nasal |
| Wj/sheep/03 | -14.3 | -0.357 | -0.197 | 1.81 | -0.03 | RNA | 40 | Nasal |
| V/revival/G | 3.6 | 0.089 | 0.05 | 1.76 | 0.01 | RNA | 40 | Nasal |
| Migwi/G3 | 1.2 | 0.03 | 0.014 | 2.18 | 0.05 | RNA | 40 | Nasal |
| Is/42/1 | 1 | 0.025 | 0.004 | 6.5 | 0.04 | RNA | 40 | Nasal |
| Is/35/1 | 1.2 | 0.03 | 0.022 | 1.34 | 0.02 | RNA | 40 | Blood |
| Is/40/1 | 2.2 | 0.056 | 0.031 | 1.8 | 0.02 | RNA | 40 | Blood |
| Is/15/1 | 1.4 | 0.036 | 0.009 | 3.9 | 0.01 | RNA | 40 | Blood |
| Is/12/1 | 1.3 | 0.034 | 0.02 | 1.66 | 0.01 | RNA | 40 | Blood |
| Is/37/1 | 7 | 0.174 | 0.04 | 4.39 | 0.01 | RNA | 40 | Blood |
| Is/36/1 | -1.1 | -0.028 | -0.025 | 1.13 | 0.06 | RNA | 40 | Blood |
| Is/23/1 | -1.3 | -0.033 | -0.019 | 1.72 | 0.07 | RNA | 40 | Blood |
| Is/18/1 | 18.1 | 0.453 | 0.325 | 1.39 | 0.33 | RNA | 40 | Blood |
| Is/31/1 | 17.4 | 0.435 | 0.288 | 1.51 | 0.24 | RNA | 40 | Blood |
| Sample X(k) | 14.6 | 0.365 | 0.252 | 1.45 | 0.65 | RNA | 40 | Blood |
| Is/41/0 | 46.2 | 1.155 | 0.861 | 1.34 | 0.24 | RNA | 40 | Blood |
| Is/19/0 | 36.3 | 0.908 | 0.647 | 1.4 | 0.18 | RNA | 40 | Blood |
| Is/27/0 | 38.3 | 0.957 | 0.691 | 1.38 | 0.21 | RNA | 40 | Blood |
| Sample Y(L) | 22.2 | 0.556 | 0.376 | 1.48 | 0.39 | RNA | 40 | Blood |
