# Supplementary material for: Peste Des Petits Ruminants (PPR) in Dromedary Camels and Small Ruminants in Mandera and Wajir Counties of Kenya
Source: Adv Virol. 2019 Mar 4;2019:4028720. doi: 10.1155/2019/4028720 (PMC6425320; doi:10.1155/2019/4028720)
Supplement: Supplementary Materials — List of tables that contain data of samples collected with their respective locations, RNA quantification, and homologous gene sequences from the NCBI used to form the phylogenetic tree. [file 4028720.f1.zip › 4028720.f1/Table 12 Homologous gene sequences in the NCBI database_AV_2677400.docx]

## *Table 10 Homologous gene sequences in the NCBI database*

KF939643.1 Dakawa_Tanzania (KF939643.1)

CCTTCCTCCAGCATAAAATAGGAGAGGGAGAGTCACATGCATCGGCGACC

AGGGAAGAAGTCAAAGCTGCGACCCCAAATGGGCCCGACGAAAAGGACAA

AACTCGGGCGCGCTCAGGAAAGCCAAGAGGAGGAACCCCCGACCAACTGC

TCCTGGAAATCATGCCTGAAGACGAGGTCCCGCGAGGGTCTGGACAAAAC

CCTCGTGAGGCTC

>Kenya_PPRV_Camel_Mandera

CCTTCCTCCAGCATAAAATAGGAGTGGGAGAGTCACATGCATCGGCGACC

AGGGAAGAAGTCAAAGCTGCGACCCCAAATGGGCCCGACGAAAAGGACAA

ATCTCGGGCGCGCTCAGGAAAGCCAAGAGGAGGAACCCCCGACCAACTGC

TCCTGGAAATCATGCCTGAAGACGAGGTCCCGCGAGGGTCTGGACAAAAC

CCTCGTGAGGCTC

>Melela_Tanzania (KT989870.1)

CCTTCCTCCAGCATAAAATAGGAGAGGGAGAGTCACATGCATCGGCGACC

AGGGAAGAAGTCAAAGCTGCGACCCCCAATGGGCCCGACGAAAAGGACAA

AACTCGGGCGCGCTCAGGAAAGCCAAGAGGAGGAACCCCCGACCAACTGC

TCCTGGAAATCATGCCTGAAGACGAGGTCCCGCGAGGTTCTGGACAAAAC

CCTCGTGAGGCTC

>Ngorongoro_Tanzania (KF939644.1)

CCTTCCTCCAGCATAAAATAGGAGAGGGAGAGTCACATGCATCGGCGACC

AGGGAAGAAGTCAAAGCTGCGACCCCAAATGGGCCCGACGAAAAGGACAA

AACTCGGGGGCGCTCAGGAAAGCCAAGAGGAGGAACCCCCGACCAACTGC

TCCTAGAAATTATGCCTGAAGACGAAGTCCCGCGAGGGTCTGGACAAAAC

CCTCGTGAGGCTC

>Ethiopia_1994(KJ67540.1)

CCTTCCTCCAGCATAAAATAGGAGAGGGAGAGTCACATGCATCGGCGACC

AGGGAAGAAGTCAAAGCTGCGACCCCAAATGGGCCCGACGAAAAGGACAA

AAAACGAGCACGCTCAGGAAGGCCAAGAGGAGGAACCCCCGACCAACTGC

TCCTGGAAATCATGCCTGAAGACGAGGTCCCGCGAGGGTCTGGACAAAAC

CCTCGTGAGGCTC

>Sudan_MIELIK_72(JN647693.1)

CCTTCCTCCAGCATAAAATAGGAGAGGGAGAGTCACATGCATCGGCGACC

AGGGAAGAAGTCAAAGCTGCGACCCCAAATGGGCCCGACGAAAAGGACAA

AAAACGAGCACGCTCAGGAAGGCCAAGAGGAGGAACCCCCGACCAACTGC

TCCTGGAAATCATGCCTGAAGACGAGGTCCCGCGAGGGTCTGGACAAAAC

CCTCGTGAGGCTC

>Nigeria 75/1 (L39878.1)

CCTTCCTCCAGCATAAAATAGGAGAGGGAGAGTCACATGCATCGGCGACC

AGGGAAGAAGTCAAAGCTGCGACCCCAAATGGGCCCGACGAAAAGGACAA

AAAACGAGCACGCTCAGGAAGGCCAAGAGGAGGAACCCCCGACCAACTGC

TCCTGGAAATCATGCCTGAAGACGAGGTCCCGCGAGGGTCTGGACAAAAC

CCTCGTGAGGCTC

>Turkana_Kenya (KM463083.1)

CCTTCCTCCAGCATAAAATAGGAGAGGGAGAGTCACATGCATCGGCGACC

GGGGAAGAAGTCAAAGCTGCGACCCCAAATGGGCCCGACGAAAAGGACAA

AACTCGGGCGCGCTCAGGAAAGCCAAGAGGAGGAACCCCTGACCAACTGC

TCCTGGAAATCATGCCTGAAGACGAGGTCCCGCGAGGGTCTGGACAAAAC

CCTCGTGAGGCTC

>Uganda_2012 (KJ867543.1)

CCTTCCTCCAGCATAAAATAGGAGAGGGAGAGTCACATGCATCGGCGACC

GGGGAAGAAGTCAAAGCTGCGACCCCAAATGGGCCCGACGAAAAGGACAA

AACTCGGGCGCGCTCAGGAAAGCCAAGAGGAGGAACCCCTGACCAACTGC

TCCTGGAAATCATGCCTGAAGACGAGGTCCCGCGAGGGTCTGGACAAAAC

CCTCGTGAGGCTC

>ETHIOPIA (JN647699.1)

CCTTCCTCCAGCATAAAATAGGAGAGGGAGAGTCACATGCATCGGCGACC

AGGGAAGAAGTCAAAGCTGCGACCCCAAATGGGCCCGACGAAAAGGACAA

AAAACGAGCACGCTCAGGAAGGCCAAGAGGAGGAACCCCCGACCAACTGC

TCCTGGAAATCATGCCTGAAGACGAGGTCCCGCGAGGGTCTGGACAAAAC

CCTCGTGAGGCTC

>Ugada_2012 (KP691481.1)

CCTTCCTCCAGCATAAAATAGGAGAGGGAGAGTCACATGCATCGGCGACC

GGGGAAGAAGTCAAAGCTGCGACCCCAAATGGGCCCGACGAAAAGGACAA

AACTCGGGCGCGCTCAGGAAAGCCAAGAGGAGGAACCCCTGACCAACTGC

TCCTGGAAATCATGCCTGAAGACGAGGTCCCGCGAGGGTCTGGACAAAAC

CCTCGTGAGGCTC

>Uganda_201 (KP691482.1)

CCTTCCTCCAGCATAAAATAGGAGAGGGAGAGTCACATGCATCGGCGACC

GGGGAAGAAGTCAAAGCTGCGACCCCAAATGGGCCCGACGAAAAGGACAA

AACTCGGGCGCGCTCAGGAAAGCCAAGAGGAGGAACCCCTGACCAACTGC

TCCTGGAAATCATGCCTGAAGACGAGGTCCCGCGAGGGTCTGGACAAAAC

CCTCGTGAGGCTC

>Comoros_Ouellah41 (KM669158.1)

CCTTCCTCCAGCATAAAATAGGAGAGGGAGAGTCACATGCATCGGCGACC

AGGGAAGAAGTCAAAGCTGCGACCCCAAATGGGCCCGACGAAAAGGACAA

AACTCGGGCGCGCTCAGGAAAGCCAAGAGGAGGAACCCCCGACCAACTGC

TCCTGGAAATCATGCCTGAAGACGAGGTCCCGCGAGGGTCTGGACAAAAC

CCTCGTGAGGCTC

>Sudan_Sinar(DQ840158.1)

CCTTCCTCCAGCATAAAATAGGAGAGGGAGAGTCACATGCATCGGCGACC

AGGGAAGAAGTCAAAGCTGCGACCCCAAATGGGCCCGACGAAAAGGACAA

AAAACGAGCACGCTCAGGAAGGCCAAGAGGAGGAACCCCCGACCAACTGC

TCCTGGAAATCATGCCTGAAGACGAGGTCCCGCGAGGGTCTGGACAAAAC

CCTCGTGAGGCTC

>Ethiopia (DQ840183.1)

CCTTCCTCCAGCACAAAATAGGAGAGGGAGAGTCACATGCATCGGCGACC

AGGGAAGAAGTCAAAGCTGCGACCCCACATGGGCCCGACGAAAAGGGCAA

AACTCGGGCACGCTCAGGAAGGCCAAGAGGAGGAACCCCCGACCAACTGC

TCTTGGAAATCATGCCTGAAGACGAGGTCCCGCGAGGGTCTGGACAAAAC

CCTCGTGAGGCTC

>China_Tibet (EU815054.1)

CCTTCCTCCAGCACCAAACAGGAGGGGGAGAGTCGTCCGCACCAGCGACC

AGAGAAGGGGTCAAAGCTGTGATCCCAAACGGATCCGAAGAAAGGGACAG

AAAGCAAACACGCCCAGGAAGGCCCAGAGGAGAGACCCCCGGCCAACTGC

TCCTGGAAATCATGCCAGAGGATGAGGTTTCGCGAGAATCTGGTCAAAAC

CCTCGTGAGGCTC

>China/33/2007 (KX421388.1)

CCTTCCTCCAGCACCAAACAGGAGGGGGAGAGTCGTCCGCACCAGCGACC

AGAGAAGGGGTCAAAGCTGCGATCCCAAACGGATCCGAAGAAAGGGACAG

AAAGCAAACACGCCCAGGAAGGCCCAGAGGAGAGACCCCCGGCCAACTGC

TCCTGGAAATCATGCCAGAGGATGAGGTTTCGCGAGAATCTGGTCAAAAC

CCTCGTGAGGCTC

>India_TN_2004 (KX860078.1)

CCTTCCTCCAGCACCAAACAGGAGGGGGAGAGTCGTCCGCACCAGCGACC

AGAGAAGGGGTCAAAGCTGCGATCCCAAACGGATCCGAAGAAAGGGACAG

AAAGCAAACACGCCCAGGAAGGCCCAGAGGAGAGACCCCCGGCCAACTGC

TCCTGGAAATCATGCCAGAGGATGAAGTCTCGCGAGAACCTGGTCAAAAC

CCTCGTGAGGCTC

>Iran_IR-ZHN336-11 (JX898862.1)

CCTTCCTCCAGCACAAAACAGGAGAGGGAGAGTCGTCCGCACCAGCAACC

AGAGAGGGGGTCAAAGCTGCGATCCCAAACGGATCCGAAGAAAGGGACAG

GAAGCAAACACGCTCAGGAAGGCCCAGAGGAGAGACCCCCAGCCAACTGC

TCCTGGAAATCATGCCAGAGGATGAGGTCTCGCGAGAGTCTGGTCAAAAC

CCTCGTGAGGCTC

>Nigeia_75/1 (KY628761.1)

CCTTCCTCCAGCATAAAACAGATGAGGGAGAGTCGCCTACACCAGCGACC

AGAGAAGAAGTCAAAGCTGCGATCCCAAATGGGTCCGAAGGAAGGGACAC

AAAGCGAACACGCTCAGGAAAGCCCAGAGGAGAAACTCCCGGCCAACTGC

TTCCGGAGATCATGCAAGAGGATGAACTCTCGCGAGAGTCTAGTCAAAAC

CCTCGTGAGGCTC

>Nigeria_75/1 (JN647715.1)

CCTTCCTCCAGCATAAAACAGATGAGGGAGAGTCGCCTACACCAGCGACC

AGAGAAGAAGTCAAAGCTGCGATCCCAAATGGGTCCGAAGGAAGGGACAC

AAAGCGAACACGCTCAGGAAAGCCCAGAGGAGAAACTCCCGGCCAACTGC

TTCCGGAGATCATGCAAGAGGATGAACTCTCGCGAGAGTCTAGTCAAAAC

CCTCGTGAGGCTC

>Kenya_KN1(KP100649.1)

CCTTCCTCCAGCATAAAATAGGAGAGGGAGAGTCACATGCATCGGCGACC

GGGGAAGAAGTCAAAGCTGCGACCCCAAATGGGCCCGACGAAAAGGACAA

AACTCGGGCGCGCTCAGGAAAGCCAAGAGGAGGAACCCCTGACCAACTGC

TCCTGGAAATCATGCCTGAAGACGAGGTCCCGCGAGGGTCTGGACAAAAC

CCTCGTGAGGCTC

>KN5/2011_Kenya(KM463083.1)

CCTTCCTCCAGCATAAAATAGGAGAGGGAGAGTCACATGCATCGGCGACC

GGGGAAGAAGTCAAAGCTGCGACCCCAAATGGGCCCGACGAAAAGGACAA

AACTCGGGCGCGCTCAGGAAAGCCAAGAGGAGGAACCCCTGACCAACTGC

TCCTGGAAATCATGCCTGAAGACGAGGTCCCGCGAGGGTCTGGACAAAAC

CCTCGTGAGGCTC

>Uganda_2012(KJ867543.1)

CCTTCCTCCAGCATAAAATAGGAGAGGGAGAGTCACATGCATCGGCGACC

GGGGAAGAAGTCAAAGCTGCGACCCCAAATGGGCCCGACGAAAAGGACAA

AACTCGGGCGCGCTCAGGAAAGCCAAGAGGAGGAACCCCTGACCAACTGC

TCCTGGAAATCATGCCTGAAGACGAGGTCCCGCGAGGGTCTGGACAAAAC

CCTCGTGAGGCTC

>Comoros_Ouellah(KM669158.1)

CCTTCCTCCAGCATAAAATAGGAGAGGGAGAGTCACATGCATCGGCGACC

AGGGAAGAAGTCAAAGCTGCGACCCCAAATGGGCCCGACGAAAAGGACAA

AACTCGGGCGCGCTCAGGAAAGCCAAGAGGAGGAACCCCCGACCAACTGC

TCCTGGAAATCATGCCTGAAGACGAGGTCCCGCGAGGGTCTGGACAAAAC

CCTCGTGAGGCTC

>Sudan_Sinar/72(DQ840158.1)

CCTTCCTCCAGCATAAAATAGGAGAGGGAGAGTCACATGCATCGGCGACC

AGGGAAGAAGTCAAAGCTGCGACCCCAAATGGGCCCGACGAAAAGGACAA

AAAACGAGCACGCTCAGGAAGGCCAAGAGGAGGAACCCCCGACCAACTGC

TCCTGGAAATCATGCCTGAAGACGAGGTCCCGCGAGGGTCTGGACAAAAC

CCTCGTGAGGCTC

>Nigeria(L39878.1)

CCTTCCTCCAGCATAAAATAGGAGAGGGAGAGTCACATGCATCGGCGACC

AGGGAAGAAGTCAAAGCTGCGACCCCAAATGGGCCCGACGAAAAGGACAA

AAAACGAGCACGCTCAGGAAGGCCAAGAGGAGGAACCCCCGACCAACTGC

TCCTGGAAATCATGCCTGAAGACGAGGTCCCGCGAGGGTCTGGACAAAAC

CCTCGTGAGGCTC

>Ethiopia(DQ840183.1)

CCTTCCTCCAGCACAAAATAGGAGAGGGAGAGTCACATGCATCGGCGACC

AGGGAAGAAGTCAAAGCTGCGACCCCACATGGGCCCGACGAAAAGGGCAA

AACTCGGGCACGCTCAGGAAGGCCAAGAGGAGGAACCCCCGACCAACTGC

TCTTGGAAATCATGCCTGAAGACGAGGTCCCGCGAGGGTCTGGACAAAAC

CCTCGTGAGGCTC

>Kenya_PPRV_Goat_Wajir

CCTTCCTCCAGCATAAAATAGGAGAGGGAGAGTCACATGCATCGGCGACC

GGGGAAGAAGTCAAAGCTGCGACCCCAAATGGGCCCGACGAAAAGGACAA

AACTCGGGCGCGCTCAGGAAAGCCAAGAGGAGGAACCCCTGACCAACTGC

TCCTGGAAATCATGCCTGAAGACGAGGTCCCGCGAGGGTCTGGACAAAAG

CCTCGAGAGGCTC

>United Arab Emirates_Dorcas/86(DQ840169.1)

CCTTCCTCCAGCATAAAACAGGAGAGGGAGAGTCACATGCATCGGTGACC

AGGGAAGAAGTCACAGCTGAGACCCCAAATGGGCCCGACGAGAAGGACAA

GAAACGAGCACGCCCAGGAAGGCCAAGAGGAGGAACCCCCGACCAACTGC

TCCTGGAGATCATGCCTGAAGACGAGGTCCCGCGAGGGCCTGGACAAACC

CCTCGTGAGGCTC

>Oman_Ibri/83(DQ840168.1)

CCTTCCTCCAGCATAAAACAGGAGAGGGAGAGTCACATGCATCGGTGACC

AGGGAAGAAGTCACAGCTGAGACCCCAAATGGGCCCGACGAGAAGGACAA

GAAACGAGCACGCCCAGGAAGGCCAAGAGGAGGAACCCCCGACCAACTGC

TCCTGGAGATCATGCCTGAAGACGAGGTCCCGCGAGGGCCTGGACAAACC

CCTCGTGAGGCTC
