# Supplementary material for: Peste Des Petits Ruminants (PPR) in Dromedary Camels and Small Ruminants in Mandera and Wajir Counties of Kenya
Source: Adv Virol. 2019 Mar 4;2019:4028720. doi: 10.1155/2019/4028720 (PMC6425320; doi:10.1155/2019/4028720)
Supplement: Supplementary Materials — List of tables that contain data of samples collected with their respective locations, RNA quantification, and homologous gene sequences from the NCBI used to form the phylogenetic tree. [file 4028720.f1.zip › 4028720.f1/Table 2. Sample collected after field investigation distribution table_AV_2677390.docx]

Table 2: Sample collected after field investigation distribution table

| Samples | Mandera | Wajir | isiolo | Marsabit |  |
| --- | --- | --- | --- | --- | --- |
| Camel blood | 7 | 6 | 8 | 0 |  |
| Camel nasal discharges | 1 | 0 | 0 | 3 |  |
| Camel ocular discharges | 1 | 2 | 0 | 0 |  |
| Sheep and goats blood | 1 | 3 | 0 | 0 |  |
| Sheep and goats nasal discharge | 1 | 2 | 0 | 0 |  |
| Sheep and goats ocular discharge | 1 | 2 | 0 | 0 |  |
| TOTALS | 12 | 15 | 8 | 3 | 38 |
