# Supplementary material for: Peste Des Petits Ruminants (PPR) in Dromedary Camels and Small Ruminants in Mandera and Wajir Counties of Kenya
Source: Adv Virol. 2019 Mar 4;2019:4028720. doi: 10.1155/2019/4028720 (PMC6425320; doi:10.1155/2019/4028720)
Supplement: Supplementary Materials — List of tables that contain data of samples collected with their respective locations, RNA quantification, and homologous gene sequences from the NCBI used to form the phylogenetic tree. [file 4028720.f1.zip › 4028720.f1/Table 3 Camels examined and Sampled in Mandera_AV_2677391.docx]

Table 3 Camels examined and Sampled in Mandera

| **Herds** | **Location** | **Animal examined** | **Animal sampled** |
| --- | --- | --- | --- |
| **1** | **Eskrito** | **10** | **0** |
| **2** | **Eskrito** | **12** | **3** |
| **3** | **Sanbur** | **10** | **0** |
| **4** | **Sanbur** | **10** | **1** |
| **5** | **Sanbur** | **10** | **2** |
| **6** | Banisa | **8** | **0** |
| **7** | Banisa | **10** | **2** |
| **8** | **Lulis** | **5** | **0** |
| **9** | **Lulis** | **5** | **1** |
| **10** | **Lulis** | **5** | **0** |
|  | **Total** | **85** | **9** |
