# Supplementary material for: Peste Des Petits Ruminants (PPR) in Dromedary Camels and Small Ruminants in Mandera and Wajir Counties of Kenya
Source: Adv Virol. 2019 Mar 4;2019:4028720. doi: 10.1155/2019/4028720 (PMC6425320; doi:10.1155/2019/4028720)
Supplement: Supplementary Materials — List of tables that contain data of samples collected with their respective locations, RNA quantification, and homologous gene sequences from the NCBI used to form the phylogenetic tree. [file 4028720.f1.zip › 4028720.f1/Table 6 Camels examined and sampled in Wajir County_AV_2677394.docx]

Table 6: Camels examined and sampled in Wajir County

| **Herds** | **Location** | **Animal examined** | **Animal Sampled** |
| --- | --- | --- | --- |
| **1** | Irigani | **5** | **0** |
| **2** | Irigani | **4** | **1** |
| **3** | Irigani | **5** | **0** |
| **4** | Bojigaras | **4** | **0** |
| **6** | Bojigaras | **7** | **2** |
| **7** | Bojigaras | **7** | **0** |
| **8** | Bojigaras | **6** | **0** |
| **9** | Ibrahim Ule | **8** | **1** |
| **10** | Ibrahim Ule | **5** | **0** |
| **11** | Ibrahim Ule | **6** | **0** |
| **12** | Leheley | **11** | **2** |
| **13** | Leheley | **13** | **2** |
| **14** | Leheley | **10** | **0** |
|  | **Total** | **90** | **8** |
