# Supplementary material for: Peste Des Petits Ruminants (PPR) in Dromedary Camels and Small Ruminants in Mandera and Wajir Counties of Kenya
Source: Adv Virol. 2019 Mar 4;2019:4028720. doi: 10.1155/2019/4028720 (PMC6425320; doi:10.1155/2019/4028720)
Supplement: Supplementary Materials — List of tables that contain data of samples collected with their respective locations, RNA quantification, and homologous gene sequences from the NCBI used to form the phylogenetic tree. [file 4028720.f1.zip › 4028720.f1/Table 7 Sheep and goats examined and sampled in Wajir County_AV_2677395.docx]

Table 7 Sheep and goats examined and sampled in Wajir County

| **Herds** | **Location** | **Animal examined** | **Animal Sampled** |
| --- | --- | --- | --- |
| **1** | Irigani | **12** | **3** |
| **2** | Irigani | **17** | **0** |
| **3** | Bojigaras | **9** | **1** |
| **4** | Leheley | **10** | **3** |
|  | **Total** | **48** | **7** |
