# Supplementary material for: Peste Des Petits Ruminants (PPR) in Dromedary Camels and Small Ruminants in Mandera and Wajir Counties of Kenya
Source: Adv Virol. 2019 Mar 4;2019:4028720. doi: 10.1155/2019/4028720 (PMC6425320; doi:10.1155/2019/4028720)
Supplement: Supplementary Materials — List of tables that contain data of samples collected with their respective locations, RNA quantification, and homologous gene sequences from the NCBI used to form the phylogenetic tree. [file 4028720.f1.zip › 4028720.f1/Table 8 Camels examined and Sampled in Isiolo County_AV_2677396.docx]

Table 8 Camels examined and Sampled in Isiolo County

| **Herds** | **Location** | **Animal examined** | **Animal sampled** |
| --- | --- | --- | --- |
| 1 | Kulamawe | 10 | 0 |
| 2 | Kulamawe | 12 | 1 |
| 3 | Kulamawe | 10 | 0 |
| 4 | Kulamawe | 10 | 1 |
| 5 | Kulamawe | 15 | 0 |
| 6 | Kulamawe | 14 | 1 |
| 7 | Kina | 26 | 2 |
| 8 | Kina | 24 | 3 |
|  | **Totals** | **131** | **8** |
