# Supplementary material for: Peste Des Petits Ruminants (PPR) in Dromedary Camels and Small Ruminants in Mandera and Wajir Counties of Kenya
Source: Adv Virol. 2019 Mar 4;2019:4028720. doi: 10.1155/2019/4028720 (PMC6425320; doi:10.1155/2019/4028720)
Supplement: Supplementary Materials — List of tables that contain data of samples collected with their respective locations, RNA quantification, and homologous gene sequences from the NCBI used to form the phylogenetic tree. [file 4028720.f1.zip › 4028720.f1/Table 9 PCR reaction master mix components_AV_2677397.docx]

*Table 9 PCR reaction master mix components*

| **No.** | **Component** | **Volume (µl)** |
| --- | --- | --- |
| 1. | 2X RT-PCR Buffer | 12.5 |
| 2. | 10µM Forward primer | 1.0 |
| 3. | 10µM Reverse primer | 1.0 |
| 4. | Nuclease-free water | 9.0 |
| 5. | 25X RT-PCR Enzyme Mix | 0.5 |
| 6. | Extracted RNA template | 1.0 |
|  | Total volume per reaction | 25.0 |
